# Antigen recognition by human γδ T cells: one step closer to knowing

**DOI:** 10.1111/imcb.12334

**Published:** 2020-04-23

**Authors:** Matthias Eberl

**Affiliations:** ^1^ Division of Infection and Immunity School of Medicine Cardiff University Cardiff UK; ^2^ Systems Immunity Research Institute Cardiff University Cardiff UK

## Abstract

Sensing of self and non‐self phosphoantigens by human Vγ9/Vδ2 T cells in the context of the butyrophilin family members BTN2A1 and BTN3A1.

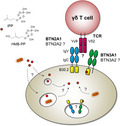

The mammalian immune system is characterized by a magnificent division of labor between lymphocyte subsets that act in concert to sense and fight the widest possible range of threats to health and life. Broadly speaking, classical CD4^+^ T cells screen extracellular spaces for the presence of foreign structures that have been captured, processed and bound to major histocompatibility complex (MHC) class II by antigen‐presenting cells. CD8^+^ T cells are in turn wired to inspect host tissues for the expression of abnormal proteins displayed in the context of MHC class I during viral infection or malignancy. Natural killer cells complement these responses by surveying the body for loss of functional MHC molecules on infected or transformed host cells.

γδ T cells pride themselves in having so far escaped the scientists’ desire to assign them a clear role within conventional schemes of immunology, not least because of their apparent lack of a general restricting element akin to MHC. Rather, evidence is mounting that in many cases these “unconventional” T cells may instead detect metabolic changes in host cells and survey tissue integrity by monitoring expression of stress‐related self‐antigens and nonpeptide molecules in the absence of orthodox antigen presentation.^1^, ^2^ Two elegant studies by Rigau *et* *al.* ^3^ and Karunakaran *et al.* ^4^ now make an important contribution toward our understanding of how a subset of human γδ T cells responds to such nonpeptide compounds.

In the vertiginous field of unconventional T‐cell biology, human Vγ9/Vδ2 T cells are strangers in a strange land. Vγ9/Vδ2 T cells typically comprise 0.5%–5% of circulating blood T cells and respond readily to both self‐ and non‐self‐metabolites, often referred to as “phosphoantigens.”^2^ By far the most potent of these is (*E*)‐4‐hydroxy‐3‐methyl‐but‐2‐enyl pyrophosphate (HMB‐PP), a microbial precursor of isopentenyl pyrophosphate (IPP), the ubiquitous building block of all higher isoprenoids. HMB‐PP is produced by the majority of Gram‐negative bacteria and bad microbes including *Mycobacterium tuberculosis*, *Clostridium difficile* and *Listeria monocytogenes*, as well as by malaria parasites and *Toxoplasma gondii*.

Humans do not produce HMB‐PP, instead generating IPP via the mevalonate pathway, one of the commercially most attractive drug targets in the body. Inhibition of β‐hydroxy β‐methylglutaryl‐coenzyme A reductase by statins abrogates downstream generation of IPP, and consequently, the synthesis of higher isoprenoids, thereby lowering cholesterol levels in individuals at risk of cardiovascular disease. Further along in the mevalonate pathway, inhibition of farnesyl pyrophosphate synthetase can be achieved with aminobisphosphonate drugs such as zoledronate, which is used in the clinic to halt excessive bone resorption in individuals with osteoporosis, multiple myeloma and bone metastasis, but also leads to the elevation of levels of upstream metabolites, including IPP, within zoledronate‐treated cells. These zoledronate‐induced metabolic troubles of a mevalonate pathway out of control are sensed by Vγ9/Vδ2 T cells and can be counteracted using statins.

The mysterious ways underlying the Vγ9/Vδ2 T‐cell responsiveness to HMB‐PP, IPP and zoledronate have long puzzled scientists.^5^ Ground‐breaking research by Rigau *et al.* ^3^ and Karunakaran *et al.* ^4^ now sheds neon lights on this phenomenon, identifying the key role of butyrophilin 2A1 (BTN2A1) in Vγ9/Vδ2 T‐cell phosphoantigen sensing (Figure 1). BTN2A1 is joining an illustrious family of only a handful of confirmed ligands for human γδ T cells that comprise diverse molecules such as endothelial protein C receptor, annexin A2 and, most recently, also the MHC‐related molecule MR1.^2^, ^6^

**Figure 1:**
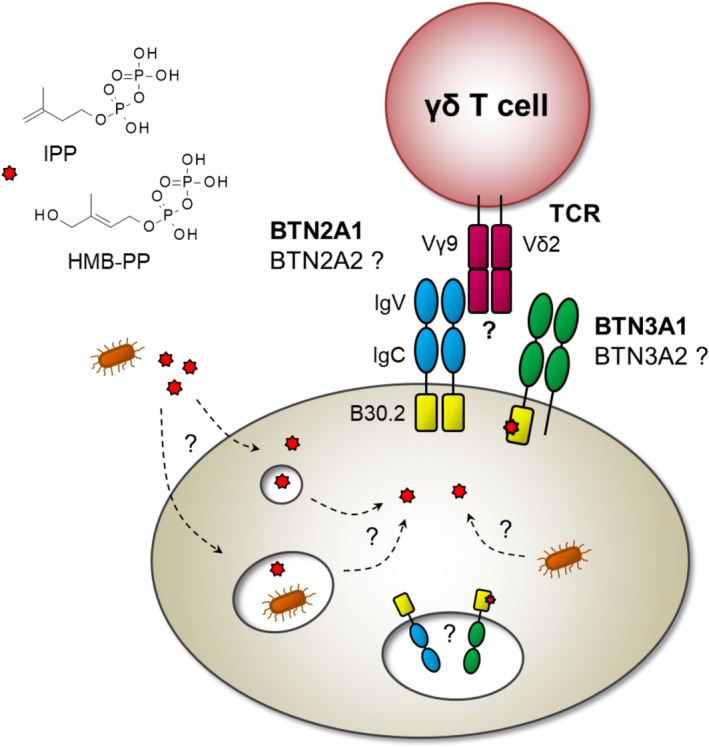
Vγ9/Vδ2 T‐cell response to microbial antigens and self‐phosphoantigens—the little things that give it away. BTN2A1 binds directly to the Vγ9 chain of the γδ TCR, possibly as homodimer, while also interacting closely with BTN3A1, which may additionally form homodimers and/or associate with BTN3A2 or BTN3A3. Whether these contacts occur simultaneously, or involve sequential interactions at another time, another place remains to be resolved as indicated by the question marks in the figure. In particular, the role of BTN2A2 is unclear at present. Phosphoantigens may reach the B30.2 domain of BTN3A1 via various pathways: intracellularly upon changes in the metabolic flux through the mevalonate pathway, for example, after zoledronate treatment, or upon release from pathogens in the cytosol or in phagocytic vesicles. Exogenous HMB‐PP and IPP may be shuttled directly into the cytosol, or be taken up by pinocytosis or endocytosis. Phosphoantigen binding to BTN3A1 may take place in the cell surface membrane or, far away so close, in a putative loading compartment inside the cell, and may be aided by other BTN3 isoforms and cofactors. Of note, baseline responses to BTN2A1 in the absence of phosphoantigens are still detectable, and the precise ligand specificity of the CDR3 regions of the Vγ9/Vδ2 TCR remains unknown. BTN2A1, butyrophilin 2A1; HMB‐PP, (*E*)‐4‐hydroxy‐3‐methyl‐but‐2‐enyl pyrophosphate; Ig, immunoglobulin; IPP, isopentenyl pyrophosphate; TCR, T‐cell receptor.

The clear implication of BTN2A1 in human Vγ9/Vδ2 T‐cell responses is a sort of homecoming for γδ T‐cell aficionados, because the related protein BTN3A1 had already been shown to be required for Vγ9/Vδ2 T‐cell responses to phosphoantigens.^7^ Other butyrophilin family members with γδ T‐cell regulatory activity include BTNL3/BTNL8 (human Vγ4^+^ T cells), Btnl1/Btnl6 (mouse Vγ7^+^ T cells) and Skint1/Skint2 (mouse Vγ5^+^ T cells).^8^ Of note, HMB‐PP and IPP were previously shown to bind to the intracellular B30.2 domain of BTN3A1,^9^ yet it has remained unclear whether and how this binding might translate to recognition of the extracellular portion of BTN3A1 via the Vγ9/Vδ2 T‐cell receptor (TCR). Additional factors have been postulated to be involved in this response, including F1‐ATPase, periplakin, RhoB and ABCA1, yet none of these acts as direct ligand for the Vγ9/Vδ2 TCR.^2^

First and foremost, the papers by Rigau *et al.* ^3^ and Karunakaran *et al.* ^4^ are a striking testimony to the power of modern techniques that have allowed scientists to propel forward like a bullet the blue sky concepts that could not easily be tested with older methodologies. Rigau *et al.* employed a genome‐wide CRISPR/Cas9 (clustered regularly interspaced short palindromic repeats/CRISPR‐associated protein 9) knockout screen of LM‐MEL‐62 melanoma cells that stained brightly with a soluble Vγ9/Vδ2 TCR tetramer, and identified *BTN2A1* as the most significant guide RNA responsible for tetramer reactivity.^3^ In support, knockdown and re‐expression experiments together with the use of anti‐BTN2A1 monoclonal antibodies confirmed a crucial role of BTN2A1 in mediating binding of the Vγ9/Vδ2 TCR tetramer, and in driving responses to HMB‐PP and zoledronate. Karunakaran *et al.* utilized an unbiased screen of radiation hybrids between *BTN3A1*‐transduced rodent cells and CHO cells carrying human chromosome 6.^10^ RNAseq analysis of positive clones mediating phosphoantigen‐dependent activation of Vγ9/Vδ2 TCR transfectants identified a 580‐kb island containing *BTN3A1*, *BTN3A2*, *BTN3A3*, *BTN2A1* and *BTN2A2* as candidates. Specific knockdown of *BTN2A1* but not *BTN2A2* numbed Vγ9/Vδ2 TCR responses to HMB‐PP and zoledronate, whereas re‐expression of *BTN2A1* restored binding of soluble Vγ9/Vδ2 TCR tetramers to *BTN2* ^−/−^ cells, thereby corroborating and extending the findings of Rigau and colleagues.

Of note, both groups report that BTN2A1 binds within seconds to Vγ9/Vδ2 as well as Vγ9/Vδ1 TCRs, and provide evidence for a direct interaction between BTN2A1 and the Vγ9 chain while ruling out contacts with the Vδ2 chain. Carefully targeted mutation of the Vγ9 chain combined with structural modeling mapped a series of key amino acids involved in this interaction, in particular R20, E70 and H85.^3^ *Vice versa*, targeted mutation of R65, R124, Y126 and E135 in the BTN2A1 protein completely abrogated binding to the Vγ9/Vδ2 TCR.^4^

So how does the discovery of BTN2A1 as a Vγ9/Vδ2 ligand square with the previous implication of BTN3A1 in phosphoantigen sensing? Well, sometimes you can't make it on your own. Both molecules appear to be essential but not sufficient on their own, and only coexpression of *BTN2A1* and *BTN3A1* elicits Vγ9/Vδ2 T‐cell responses to HMB‐PP or zoledronate. And despite both molecules possessing intracellular B30.2 domains, phosphoantigens appear to only bind to the B30.2 domain of BTN3A1 but not to that of BTN2A1.^3^ Using confocal microscopy and Förster resonance energy transfer analyses, Rigau *et al.* in fact demonstrate that BTN2A1 and BTN3A1 colocalize at the cell surface and that the two molecules associate intracellularly and extracellularly.^3^ These results are complemented by crosslinking and immunoprecipitation studies by Karunakaran *et al.* using a 16‐Å spacer, indicating a close and possibly direct association of BTN2A1 with BTN3A1 on the cell surface.^4^

The model emerging from these findings suggests that BTN2A1 exclusively touches the germline‐encoded HV4 region of the Vγ9 chain, with no involvement of the Vδ2 chain, whereas phosphoantigen specificity of the TCR is conferred by BTN3A1 recognition via both the CDR3 region of the Vγ9 chain and the CDR2 region of the Vδ2 chain.^3^, ^4^ But is that all? The potential contribution of the other members of the BTN2/3 family, and whether target recognition occurs with or without them, remains elusive. Previous reports suggested that BTN3A2 and/or BTN3A3 may help facilitate surface expression of BTN3A1 and form heterodimers. Here, BTN3A2 was not required for the phosphoantigen‐dependent activation of Vγ9/Vδ2 T cells by *BTN2A1*–*BTN3A1* co‐transfected hamster cells, but nevertheless enhanced the response moderately.^3^ In addition, BTN2A2 appears to be capable of binding to the Vγ9 chain with similar affinity to BTN2A1, with as yet unknown relevance.^4^ To walk on, we will require further biochemical and structural work into the precise contribution of the different BTN2 and BTN3 isoforms, the role of the B30.2 domains and their discrimination between self‐ and non‐self phosphoantigens, the vesicular trafficking pathways that shuttle phosphoantigens to the cytosol, and characterization of the intracellular and extracellular partner molecules involved (Figure 1).

The implications of these new findings are manifold and light the way for future investigations. For instance, it is tempting to speculate that BTN2A1 and/or BTN3A1 may play a role in the exit of positively selected mature Vγ9/Vδ2 T cells from the thymus. Moreover, given that BTN2A1–BTN3A1 complexes can be formed independently of phosphoantigens and that BTN2A1 elicits a relatively small but discernible baseline response on its own,^5^ there is a light for the existence of a further, true antigen that is recognized by the Vγ9/Vδ2 TCR in a CDR3‐mediated fashion but has remained invisible so far. Intriguingly, such a hypothetical ligand/cofactor must be conserved evolutionarily—*BTN2A1*–*BTN3A1*‐transduced mouse and hamster cells are targeted readily by human Vγ9/Vδ2 TCRs,^3^, ^4^ and for the first time a nonprimate original of the species, the alpaca (*Vicugna pacos*), has been reported to also possess a phosphoantigen‐reactive Vγ9/Vδ2 T‐cell subset.^11^ Finally, given the strong translational and applied interest in Vγ9/Vδ2 T cells, BTN2A1 is a highly relevant target candidate for the development of agonistic and/or antagonistic miracle drugs in the context of microbial infection, autoimmunity and tumor immunotherapy.

The seminal publications by Rigau *et al.* and Karunakaran *et al.* on BTN2A1 as a novel ligand for human Vγ9/Vδ2 T cells extend previous reports of similar γ‐chain‐specific, superantigen‐like interactions of other butyrophilin family members with γδ T cells in humans and in mice, evoking Susumu Tonegawa’s unforgettable prediction that γδ T cells “may be involved in an entirely new aspect of immunity,”^12^ clearly distinct from classical CD4^+^ and CD8^+^ T cells and MHC‐restricted adaptive immunity. Yet one caveat remains. We may be one step closer to knowing but with regard to understanding the antigen specificity of the γδ TCR CDR3 region we still haven't found what we are looking for.

## Conflict of Interest

I declare no conflicts of interest.
